# Supplementary material for: HIVprotI: an integrated web based platform for prediction and design of HIV proteins inhibitors
Source: J Cheminform. 2018 Mar 9;10:12. doi: 10.1186/s13321-018-0266-y (PMC5845081; doi:10.1186/s13321-018-0266-y)
Supplement: Supplementary file 2 — Additional file 2. Source code of HIVProtI web server. [file 13321_2018_266_MOESM2_ESM.zip › HIVprotI_Source-code/hivprotI/weka-3-6-6/doc/weka/gui/sql/event/QueryExecuteEvent.html]

QueryExecuteEvent


---


|  |  |  |
| --- | --- | --- |
| **Overview** \| **Package** \| **Class** \| **Tree** \| **Deprecated** \| **Index** \| **Help** \| **Weka's home** | |  |
| **PREV CLASS**   **NEXT CLASS** | **FRAMES**    **NO FRAMES**     **All Classes** |  |
| SUMMARY: NESTED \| FIELD \| CONSTR \| METHOD | DETAIL: FIELD \| CONSTR \| METHOD |  |


---


## weka.gui.sql.event Class QueryExecuteEvent

```
java.lang.Object
  java.util.EventObject
      weka.gui.sql.event.QueryExecuteEvent
```

**All Implemented Interfaces:**: java.io.Serializable

---

``` public class QueryExecuteEvent extends java.util.EventObject ```

An event that is generated when a query is executed.

**Version:**
:   $Revision: 7059 $

**Author:**
:   FracPete (fracpete at waikato dot ac dot nz)

**See Also:**: `QueryExecuteListener`, Serialized Form

---

**Constructor Summary**

`QueryExecuteEvent(java.lang.Object source,
DbUtils utils,
java.lang.String query,
int rows,
java.sql.ResultSet rs,
java.lang.Exception ex)`
  
          constructs the event


| **Method Summary** | |
| --- | --- |
| `boolean` | `failed()`             is TRUE in case the exception is not NULL, i.e. |
| `DbUtils` | `getDbUtils()`             returns the DbUtils instance that was executed the query |
| `java.lang.Exception` | `getException()`             returns the exception, if one happened, otherwise NULL |
| `int` | `getMaxRows()`             returns the maximum number of rows to retrieve. |
| `java.lang.String` | `getQuery()`             returns the query that was executed |
| `java.sql.ResultSet` | `getResultSet()`             returns the resultset that was produced, can be null in case the query failed |
| `boolean` | `hasResult()`             whether a ResultSet was produced, e.g. |
| `java.lang.String` | `toString()`             returns the event in a string representation |

**Methods inherited from class java.util.EventObject**

`getSource`

**Methods inherited from class java.lang.Object**

`equals, getClass, hashCode, notify, notifyAll, wait, wait, wait`

**Constructor Detail**

### QueryExecuteEvent

```
public QueryExecuteEvent(java.lang.Object source,
                         DbUtils utils,
                         java.lang.String query,
                         int rows,
                         java.sql.ResultSet rs,
                         java.lang.Exception ex)
```

:   constructs the event

    **Parameters:**: `source` - the source that generated this event: `utils` - the DbUtils instance that connected to the DB: `query` - the query that is the basis for the resultset: `rows` - the maximum number of rows to retrieve (0 for all): `rs` - the ResultSet that was produced (depending on the type of SQL query it can also be NULL): `ex` - in case an exception occurred


**Method Detail**

### getDbUtils

```
public DbUtils getDbUtils()
```

:   returns the DbUtils instance that was executed the query

---


### getQuery

```
public java.lang.String getQuery()
```

:   returns the query that was executed

---


### getMaxRows

```
public int getMaxRows()
```

:   returns the maximum number of rows to retrieve. 0 means all.

---


### failed

```
public boolean failed()
```

:   is TRUE in case the exception is not NULL, i.e. the query failed

---


### hasResult

```
public boolean hasResult()
```

:   whether a ResultSet was produced, e.g. DDL commands like delete, drop
    or update do not produce one.

---


### getResultSet

```
public java.sql.ResultSet getResultSet()
```

:   returns the resultset that was produced, can be null in case the query
    failed

---


### getException

```
public java.lang.Exception getException()
```

:   returns the exception, if one happened, otherwise NULL

---


### toString

```
public java.lang.String toString()
```

:   returns the event in a string representation

    :   **Overrides:**: `toString` in class `java.util.EventObject`
    :   **Returns:**: the event in a string representation


---


|  |  |  |
| --- | --- | --- |
| **Overview** \| **Package** \| **Class** \| **Tree** \| **Deprecated** \| **Index** \| **Help** \| **Weka's home** | |  |
| **PREV CLASS**   **NEXT CLASS** | **FRAMES**    **NO FRAMES**     **All Classes** |  |
| SUMMARY: NESTED \| FIELD \| CONSTR \| METHOD | DETAIL: FIELD \| CONSTR \| METHOD |  |


---
